# Celiac Disease Prevention

**DOI:** 10.3389/fped.2018.00368

**Published:** 2018-11-30

**Authors:** Caroline Meijer, Raanan Shamir, Hania Szajewska, Luisa Mearin

**Affiliations:** ^1^Deptartment of Pediatrics, Leiden University Medical Center, Willem Alexander Children's Hospital, Leiden, Netherlands; ^2^Institute for Gastroenterology, Nutrition and Liver Diseases, Schneider Children's Medical Center, Sackler Faculty of Medicine, Tel Aviv University, Tel Aviv, Israel; ^3^Department of Pediatrics, The Medical University of Warsaw, Warsaw, Poland

**Keywords:** celiac disease, prevention, preventive strategies, environmental factors, tertiary prevention

## Abstract

Celiac disease (CD) is a common autoimmune disorder induced by ingestion of gluten in genetically susceptible individuals. Despite the prerequisite for a genetic predisposition, only a minority of the 40% of the Caucasian population that has this genetic predisposition develops the disease. Thus, environmental and/or lifestyle factors play a causal role in the development of CD. The incidence of CD has increased over the last half-century, resulting in rising interest in identifying risk factors for CD to enable primary prevention. Early infant feeding practices have been suggested as one of the factors influencing the risk of CD in genetically susceptible individuals. However, recent large prospective studies have shown that neither the timing of gluten introduction nor the duration or maintenance of breastfeeding influence the risk of CD. Also, other environmental influences have been investigated as potential risk factors, but have not led to primary prevention strategies. Secondary prevention is possible through early diagnosis and treatment. Since CD is significantly underdiagnosed and a large proportion of CD patients are asymptomatic at the time of diagnosis, secondary prevention will not identify all CD patients, as long as mass screening has not been introduced. As following a gluten-free diet is a major challenge, tertiary prevention strategies are discussed as well.

## Introduction

The incidence and prevalence of celiac disease (CD) have risen over time; this is, in part, due to the current awareness in combination with the advent of highly sensitive and specific serological tests, but it also reflects a true increase in the prevalence of CD (1, 2). The clinical presentation of CD has changed dramatically in the last decades. Patients with atypical or non-specific symptoms often report a delay in diagnosis of CD that may last for years (3) or even worse, CD remains unrecognized and, therefore, untreated (4–6). Untreated disease is associated with long-term complications, such as chronic anemia, delayed puberty, neuropsychiatric disturbances, associated autoimmune disorders, infertility, small-for-date-births, osteoporosis, and, rarely, malignancy and it can reduce the quality of life (7–9). Treatment with a gluten-free diet (GFD) reduces the burden of morbidity and mortality associated with untreated CD. Thus, prevention would be beneficial (10). Prevention is defined as any activity that reduces the burden of mortality or morbidity from disease, taking place at the primary, secondary, or tertiary level (11) (Table 1). The purpose of this review is to present the current knowledge of the preventive strategies for CD (Table 2).

**Table 1:** Definition of levels of prevention.

**Primary**	**Secondary**	**Tertiary**
Avoiding the development of a disease	Early detection and treatment	Reducing the impact of existing disease by improved treatment

**Table 2:** Some possible prevention strategies for celiac disease, as discussed in this review.

**Primary**	**Secondary**	**Tertiary**
•Infant feeding -Breastfeeding -Breastfeeding at the Time of gluten introduction -Timing of gluten introduction -Amount of gluten at the Time of gluten introduction •(Intestinal) infections •Type of delivery •Antibiotics •Microbiota	•Case finding •Screening of high-risk groups •Mass screening	•Optimal adherence to the gluten-free diet •Gluten immunogenic peptides •Dietary interview •Dietary questionnaires •Serology/duodenal biopsies •Additional treatments -Larazotide acetate -Endopeptidases -Desensitization therapy

## Primary prevention

### Infant feeding

Theoretically, CD could be prevented by avoiding gluten introduction into the feeding of infants genetically predisposed to CD. However, this is not a realistic strategy, because the strongest genetic predisposing factors for CD, HLA DQ2 and/or HLA-DQ8, are present in about 40% of the Caucasian population. In addition, most of these individuals do not develop CD, since the prevalence of CD is ~1%. Another reason why avoiding gluten ingestion by a large part of the population is not desirable is that gluten-containing cereals (among others wheat, barley and rye) are important sources of dietary iron, fiber, calcium, folate, and vitamin B12 (12, 13).

Much knowledge about the possible relationship between infant feeding practices and the development of CD has been obtained from “The Swedish epidemic of CD” during the mid-1980s. Between 1985 and 1987, the incidence of CD in Swedish children younger than 2 years of age increased 4-fold, followed by a rapid decline in its incidence around 1995 (14). The occurrence of the epidemic was related to new dietary recommendations: delaying the introduction of all gluten-containing foods to infants until 6 months of age and changes in breastfeeding practices. In Sweden, the incidence of CD diminished when earlier introduction of gluten (>4 months) was reintroduced (14). Many retrospective studies have investigated this hypothesis that delayed introduction of gluten leads to CD with conflicting results. Results of observational studies suggested the existence of a “window of opportunity” for primary prevention, by introducing gluten between 4 and 6 months of age to reduce the risk of CD (Table 3). This and other early feeding practices, such as breastfeeding and breastfeeding at the time of gluten introduction, have been investigated as primary prevention strategies for reducing the risk of CD as well (Tables 4, 5). A systemic review and meta-analysis, which included all of the studies published on this topic between 1966 and 2004, found that breastfed children had a 52% reduction in the risk of being affected by CD compared to those who were not breastfed during the time of gluten introduction (pooled OR 0.48; 95% CI: 0.40 to 0.59) (37). However, all of these studies were observational and retrospective.

**Table 3:** Evidence of the effect of the timing of gluten introduction into the diet of young children and the risk of celiac disease.

**First author, year, study**	**Conclusion**
**INTERVENTIONAL STUDIES**	
Vriezinga et al. (15), PREVENTCD	No significant difference in CD development at 3 years for gluten introduction at 4 vs. 6 months^∧^
Lionetti et al. (16), CELIPREV	No significant difference in CD development at 5 years for gluten introduction at 6 vs. 12 months
Sellitto et al. (17)	No significant difference in CDA^*^ risk for gluten introduction at 6 vs. 12 months
Hummel et al. (18)/Beyerlein et al. (19)^**^	No significant difference in CD and CDA for different gluten introduction at 6 vs. 12 months
**PROSPECTIVE COHORT STUDIES**	
Andrén Aronsson et al. (20), TEDDY	No significant difference in CD and CDA for gluten introduction at < 17 vs. 17–26 vs. >26 weeks
Jansen et al. (21), Generation R	No significant difference in CDA for gluten introduction at < 6 vs. >6 months
Størdal et al. (22), MoBa	Borderline significant difference in CD development at gluten introduction < 6 vs. >6 months
Welander et al. (23), ABIS	No significant difference in CD for different times of gluten introduction from 0 to 12 months
Norris et al. (24), DAISY	Significantly more CD with gluten introduction < 3 or >7 months vs. gluten introduction between 4 and 6 months.
Ziegler et al. (25), BABYDIAB	No significant difference in CD for gluten introduction ≤ 3 vs. >6 months
Hummel et al. (26), BABYDIAB	No significant difference in CDA for gluten introduction < 3 vs. >3 months
**RETROSPECTIVE STUDIES**	
Ivarsson et al. (27)	Significantly more CD with gluten introduction >6 months compared to gluten introduction between 4 and 6 months.
Peters et al. (28)	No significant difference in CD gluten introduction at ≤ 3 vs. >3 months
Falth-Magnusson et al. (29)	No significant difference in CD for different times of gluten introduction
**CROSS-SECTIONAL STUDY**	
Ivarsson et al. (30), ETICS	Significantly more CD with gluten introduction >6 months compared to gluten introduction between 4 and 6 months.

*CD, celiac disease; CDA, celiac disease autoimmunity*.

* *Celiac disease autoimmunity*.

** *Same population*.

∧ *Months of age*.

**Table 4:** Most important studies on the evidence of protection from celiac disease with breastfeeding.

**First author, year, study**	**Conclusion**
**INTERVENTIONAL STUDIES**	
Vriezinga et al. (15), PREVENTCD	No effect
Lionetti et al. (16), CELIPREV	No effect
**PROSPECTIVE STUDIES**	
Jansen et al. (21), Generation R	No effect
Størdal et al. (22), MoBa	No effect^*^
Welander et al. (23), ABIS	No effect
Norris et al. (24), DAISY study	No effect
Ziegler et al. (25)/Hummel et al. (26)^**^, BABYDIAB	No effect
**RETROSPECTIVE STUDIES**	
Decker et al. (31)	No effect
Roberts et al. (32)	No effect
Ivarsson et al. (27)	Protective
Peters et al. (28)	Protective
Greco et al. (33)	Protective
Ascher et al. (34)	No effect
Falth-Magnusson et al. (29)	Protective
Auricchio et al. (35)	Protective
**CROSS-SECTIONAL STUDY**	
Ivarsson et al. (30), ETICS	Protective

* *Breastfeeding (BF) > 1 year predisposing*;

** *Same population*.

**Table 5:** Evidence of the effect of breastfeeding at the time of gluten introduction and risk for celiac disease.

**First author, year, study**	**Conclusion**
**INTERVENTIONAL STUDIES**	
Vriezinga et al. (15), PREVENTCD	No effect
Lionetti et al. (16), CELIPREV	No effect
**PROSPECTIVE COHORT STUDIES**	
Andrén Aronsson et al. (36), TEDDY	No effect
Størdal et al. (22), MoBa	No effect
Hummel et al. (26), BABYDIAB	No effect
Norris et al. (24), DAISY	No effect
**RETROSPECTIVE STUDIES**	
Ivarsson et al. (27)	Protective
Peters et al. (28)	Protective
Ascher et al. (34)	No effect
Falth-Magnusson et al. (29)	Protective

Among the prospective studies that have been published, there are two gluten interventional ones, namely PREVENTCD and CELIPREV (15, 16) (Table 3):

- PREVENTCD is a multinational, randomized, double-blind, placebo-controlled dietary interventional study involving 944 children who had at least 1 first-degree relative with CD and HLA-DQ2 and/or DQ8. From age 4 to 6 months, 475 participants received 100 mg of vital gluten daily and 469 received placebo. After 24 weeks, intake of gluten was liberalized in both groups. CD serology was measured periodically. Children with elevated levels of CD antibodies and/or with symptoms suggestive of CD were offered small bowel biopsies to confirm the diagnosis. The results showed no significant difference between the groups receiving the early gluten intervention or placebo in the risk of developing CD at the age of 3 years.
- CELIPREV is an Italian multicenter, randomized, interventional study that compared early (at 6 months of age; *n* = 297) and delayed (at 12 months of age; *n* = 256) introduction of gluten into the diet of infants at risk for CD (first-degree relative with CD; HLA-DQ2 and/or DQ8 positivity). The results showed a reduced risk of developing CD by the age of 2 years in those with delayed introduction to gluten at 12 months, but no difference between groups in the risk of developing CD at 5 years of age.

A few of the large, prospective, observational cohort (non-interventional) studies assessing the relationship between infant feeding practices and the risk of CD and/or CD autoimmunity (CDA) pointed out the following (Tables 3–5):

- The Generation R cohort study, including 1679 genetically susceptible CD children from the general population of Rotterdam, the Netherlands, showed that neither breastfeeding for 6 months or longer nor later exposure to gluten (>6 months) compared to earlier exposure (< 6 months) was significantly associated with CDA (21).
- The Norwegian Mother and Child Cohort Study (MoBa) showed that breastfeeding longer than 12 months was associated with a higher risk of CD (22). However, this cohort only considered children with clinically diagnosed CD, so probably missed an important proportion of the children with CD.
- The BABYDIAB, a German cohort study, found no association between the duration of breastfeeding nor gluten introduction before or after 3 months of age and risk of CDA (26).
- The Environmental Determinants of Diabetes in the Young (TEDDY) project is an observational, prospective, cohort study that followed children at genetic risk for type 1 diabetes, wherein development of CD is a secondary outcome. The TEDDY study included 6,403 children with a genetic predisposition to developing CD in the United States, Finland, Germany, and Sweden. The study found that gluten introduction before 17 weeks of age or later than 26 weeks of age was not associated with an increased risk for CDA or CD; however, continuation of breastfeeding more than 1 month after gluten introduction compared with discontinuation of breastfeeding prior to gluten introduction was associated with increased risk of CDA but not of CD (20).

Two systematic reviews and meta-analyses, which included the above prospective interventional studies and large cohort studies (Tables 3–5), concluded that the timing of gluten introduction and the duration or maintenance of breastfeeding do not influence the development of CD (38, 39).

Interest in the quantity of gluten at introduction into the diet of infants was also raised based on the results of the Swedish CD epidemic. The evaluation of results of one retrospective observational study indicated that large amounts of gluten (>16 g/day) at the time of first introduction increased the risk of CD (27). The same group of investigators further compared, in the ETIC project, 2 populations born in 1993 and 1997; they found a lower risk of CD in the population born in 1997 who ingested significantly less gluten-containing cereal compared to the population born in 1993 (24 vs. 38 g/day intake, respectively, under the age of 2 years) (30). Also, the Swedish case control study from the TEDDY cohort, in which gluten intake was assessed by dietary questionnaires, found that a high intake (>5.0 g/day) of gluten during the first 2 years of life was associated with an increased risk of CD (36). However, a similar analysis of the data in the international PREVENTCD study showed that the amount of gluten consumed at 11–36 months of age did not influence the risk for CD development (40). Thus, the influence of the amount of gluten intake on CD risk remains a topic of discussion.

In accordance with the results from the above-mentioned studies, ESPGHAN has updated its guidelines for gluten introduction into the diet of young children. The current recommendation no longer suggests introducing gluten between 4 and 6 months of age; rather they recommend that gluten may be introduced into the infant's diet anytime between 4 and 12 completed months of age, since gluten introduction in these infants does not seem to influence the absolute risk of developing CDA or CD during childhood (38).

In addition to gluten and breastfeeding, other environmental factors may be involved in the risk and/or prevention of CD. Identifying and influencing these factors may lead to preventive strategies. Some of these factors are discussed below.

### (Intestinal) infections

Intestinal infections might change gut permeability and lead to the passage of immunogenic gluten peptides through the epithelial barrier, and thus, activate an autoimmune reaction. Many groups have studied the relationship between infections, both viral and bacterial, and the risk of CD, with varying results (Table 6). The role of early infections was retrospectively explored in the Swedish population-based incident case referent ETICS study. Having three or more parental-reported infections, regardless of the type of infection, during the first 6 months of life was associated with significantly increased risk of CD, even after adjusting for infant feeding and socioeconomic status (61).

**Table 6:** Some of the most relevant studies^#^ on infections and the risk of celiac disease or celiac disease autoimmunity.

**First author, year, study**	**Pathogen**	**Association between CD(A)**
**PROSPECTIVE STUDIES**		
Stene et al. (41)	Rotavirus	Positive
Thevenot et al. (42)	Hepatitis C virus	None
Gravina et al. (43)	Hepatitis C virus	None
Jansen et al. (44)	EBV, CMV and HSV-1	Negative
Karhus et al. (45)	Influenza	None
Dore et al. (46)	Helicobacter Pylori	None
**RETROSPECTIVE STUDIES**		
Lahdeaho et al. (47)	Adenovirus 12/40	Positive
Vesy et al. (48)	Adenovirus 12, CMV, HSV	None
Kagnoff et al. (49)	Adenovirus 12 Adenovirus 18/echovirus 11	Positive None
Mahon et al. (50)	Adenovirus 12	None
Fine et al. (51)	Hepatitis C virus	Positive
Carlsson et al. (52)	Enterovirus	None^*^
Villalta et al. (53)	Hepatitis C virus	Positive
Ruggeri et al. (54)	Hepatitis C virus	Positive
Sarmiento et al. (55)	Enterovirus, EBV, CMV, Hepatitis C virus	Positive
Tjernberg and Ludvigsson (56)	RSV	Positive
Abid et al. (57)	Hepatitis B virus	Positive
Tarish et al. (58)	Adenovirus	None
Alaedini et al. (59)	Borrelia	None
Bouziat et al. (60)	Reovirus	Positive

*CD, celiac disease; CDA, celiac disease autoimmunity; EBV, Epstein Barr virus; CMV, cytomegalovirus; HSV, herpes simplex virus; RSV, respiratory syncytial virus*.

# *Case reports were excluded*.

* *Between these infection during pregnancy and CD development in the offspring*.

Results of prospective studies are contradictory. Data from the PREVENTCD study showed no significant difference in the cumulative incidence of CD between children with and without parental-reported gastrointestinal infections in the first 18 months of life (15). However, the TEDDY study found that parental-reported early gastrointestinal infections increased the risk of CDA within the following 3 months (HR 1.33; 95% CI 1.11–1.59). This effect was observed particularly in those children with non-HLA-DQ2 genotypes who had been breastfed for < 4 months, as well as in children born in winter and introduced to gluten before the age of 6 months (62). In the prospective MoBa study, children with ≥10 infections (respiratory and gastrointestinal) before 18 months of age had a higher risk of being clinically diagnosed with CD compared with children who had ≤ 4 infections, even after adjustments for antibiotic exposure (63). Viral infections have been suggested to play a role in the development of CD (Table 9), and recently, reovirus has been reported as a trigger for the disease, both *in vitro* as well as *in vivo* (60). *In vitro*, reovirus infection induced a disruption of intestinal immune homeostasis and initiated loss of oral tolerance and T-helper inflammatory immunity to dietary antigens. In CD patients anti-Reovirus antibodies were significantly overrepresented in comparison to health controls.

However, this disruption of the immune homeostasis may not be exclusive to reovirus and their role in the development of CD should be studied prospectively.

### Type of delivery

The mode of delivery (vaginal or cesarean section [C-section]) has a strong influence on shaping the initial gut microbiota composition. It has been hypothesized that infants born by C-section acquire different bacterial communities compared to vaginally delivered infants (64), which may influence the short- and long-term immune responses to environmental factors, thereby predisposing to autoimmunity (65). Also, the type of C-section, emergency vs. elective, has been hypothesized as a different possible influencing factor, since the cord blood immune cell phenotypes are affected by stress during vaginal delivery and this does not happen by elective C-section (66). In addition, infants born vaginally and during emergency C-section are colonized at first by fecal and vaginal bacteria of the mother, whereas infants born through elective cesarean delivery are exposed initially to bacteria originating from the hospital environment and healthcare workers. Infants born by cesarean delivery are characterized by a more slowly diversifying microbiota, with a substantial absence of Bifidobacteria species and Bacteroides and the presence of facultative anaerobes, such as Clostridium species. These differences might influence the development of the mucosal immune system, the establishment of a stable intestinal host-microbial homeostasis, as well as the mucosal barrier function and ultimately contribute to the risk of acquiring immune-mediated diseases, such as CD, later in life (67).

Some studies have identified C-section as a risk factor for the development of CD (68, 69). However, more recent prospective studies have found no association (70–73) (Table 7). Recently, a large, observational, register-based, cohort study investigated the association between the type of delivery and the risk of developing CD in two independent population cohorts (Denmark, birth cohort 1995–2010 and Norway, birth cohort 2004–2012) (74). A total of 3,314 children were diagnosed with CD. C-sections were performed in 286,640 children, and the mode of delivery was not associated with an increased risk of diagnosed CD.

**Table 7:** Some of the most relevant studies on type of delivery and the risk for celiac disease.

**First author, year, study**	**Conclusion**
Koletzko et al. (73), TEDDY	No association with CDA or CD
Dydensborg Sander et al. (74), ETICS	No association with CD
Lionetti et al. (72), CELIPREV	No association with CD
Kristensen and Henriksen (68)	Positive association between emergency CS and CD
Emilsson et al. (70), MoBa	No association between CS and CD
Sevelsted et al. (71)	No association with CD
Marild et al. (69)	Positive association with CD
Decker et al. (31)	Positive association with CD
Roberts et al. (32)	Negative association between CS and CD

*CD, celiac disease; CDA, celiac disease autoimmunity; CS, cesarean section*.

In the above-mentioned Danish cohort, the association between elective C-section and diagnosed CD was positive and reached borderline statistical significance after adjusting for year of birth, sex, maternal age, education, parity, gestational age, and weight for gestational age (OR: 1.20; 95% CI: 1.00–1.43). However, this finding was not replicated in the corresponding Norwegian cohort (OR: 0.96; 95% CI: 0.79–1.17) (74). Analysis of the data from the Swedish Medical Birth Register between 1973 and 2008, comparing cases with villous atrophy with age- and sex-matched controls from the general population, found a weak association between an elective C-section and CD in offspring (adjusted odds ratio [OR] = 1.15), but no increased risk for CD diagnoses after an emergency (adjusted OR = 1.02) or any C-section (adjusted OR = 1.06) (69). Data from a population- and national register-based cohort including all children born in Denmark from January 1997 to December 2012 showed the opposite: children delivered by emergency C-section were at an increased risk for CD (adjusted OR = 1.22), whereas children delivered by elective C-section were not (adjusted OR = 0.69) (68). Thus, despite the plausible hypothesis that mode of delivery affects risk of CD, the current literature showed no association between the type of delivery and the risk of CD (Table 7).

### Antibiotics

The ETIC study found no evidence of increased CD risk with antibiotic use in the first 6 months of life (61). However, other 2 retrospective studies have shown a positive association between antibiotic use and CD risk (75, 76). A recent analysis of the TEDDY study showed that cumulative exposure to β-lactam or macrolide antibiotics, up to 6 months, during the first or second year of life and within 6 months before the seroconversion period, was not associated with CDA. Also, maternal use of antibiotics during pregnancy was also evaluated as a risk factor and did not significantly contribute to CDA risk in this study. In conclusion, the role of antibiotics in the development of CD is a topic that remains unclear and requires more research.

### Microbiota

CD development has also been linked to alterations in the human gut microbiome, which is necessary for proper development of the immune system and establishment of oral tolerance in early life (65). The contributing role of perturbations in the gut microbiota, and of specific enteric bacteria, to gluten-induced immunopathology has been shown in animal models (77). PROFICEL, a prospective study of 164 healthy Spanish newborns with a first-degree relative with CD and HLA-DQ2 and/or DQ8 positivity, reported an association between the HLA-DQ genotype and the intestinal microbiota composition. In this study, the HLA-DQ2/8 genotype and the type of feeding (breastfeeding or formula) were shown to influence, in conjunction, the composition of the intestinal microbiota (78). The high-risk genotype for developing CD (HLA-DQ2, including homozygous HLA-DQ2.5 or heterozygous DQ2.5/DQ2.2 and DQ2.2/DQ7.5) was associated with reduced numbers of Bifidobacterium, specifically of the species *B. Longum*, compared to the rest of the lower-risk genotypes (79). Also, other studies have found similar results; the duodenal and fecal microbiota of CD patients is unbalanced, with decreased numbers of anti-inflammatory bacteria, such as Bifidobacterium spp. and increased numbers of Bacteroides spp., which are only partially normalized after a long-term gluten free diet (GFD) (80–82). In a double-blind, randomized, placebo-controlled, interventional trial performed in children with newly diagnosed CD, children were randomized to receive *Bifidobacterium longum* or placebo in conjunction with a GFD (83). A decrease in both the numbers of the Bacteroides fragilis group and the fecal secretory IgA concentration was found, which might further confirm the role of microbiota in the pathogenesis of CD. But, so far, studies have failed to find a distinct microbiota profile in patients with CD.

A sub-study of the PROFICEL project, including 10 CD cases and 10 matched controls, suggests altered early proportions of Firmicutes and members of the Actinobacteria phylum (*B. Longum*) in children who later progressed to CD (84). Hopefully, the results of the Celiac Disease Genomic, Environmental, Microbiome, and Metabolomic (CDGEMM) study, a multicenter, longitudinal study of infants at risk for CD, will provide an answer to the question regarding the role of the gut microbiome and the risk of CD (85). CDGEMM aims to enroll 500 infants aged 0–6 months with a first-degree family member with CD. Health status, anthropometrics, nutritional information, household and environmental information, and blood and stool samples are being collected regularly to understand the role of the gut microbiome as an additional factor that may play a key role in early steps involved in the development of autoimmune disease (85).

In conclusion, in the field of primary prevention, infant feeding practices have been explored by interventional studies with long-term follow up, but have shown no protection for risk of CD. Other possible influences on the development of CD, especially the role of infections and the gut microbiome, need further research.

**Table:** Text box

**Summary of evidence of effectiveness of possible primary prevention strategies for celiac disease**	**Conclusion**
Infant feeding
Breastfeeding	No effect
Breastfeeding at the time of gluten introduction	No effect
Timing of gluten introduction	No effect
Amount of gluten at the time of gluten introduction	Unclear
(Intestinal) infections	Unclear
Type of delivery	No effect
Antibiotics	Unclear
Microbiota	Unknown

## Secondary prevention

### Case finding

Secondary prevention focuses on early detection and treatment (Table 8). Active case finding refers to the liberal diagnostic testing of subjects with CD-associated symptoms. In the general population, this approach has led to the early diagnosis of many patients, resulting in significant health improvement after treatment, good compliance with the GFD, and good CD-related quality of life (86, 87); unfortunately, however, it does not counter the entire under-diagnosis of CD (88, 89). Only a small proportion of the undiagnosed patients are detected with this strategy, since ~50% of the children in screening-detected studies have symptoms at the time of diagnosis (15, 16, 90).

**Table 8:** Secondary prevention strategies for celiac disease.

Case finding
Screening in high-risk groups
First-degree relatives of CD patients
Type 1 diabetes mellitus
Autoimmune thyroid disease
Autoimmune liver disease
Syndrome: Down, Turner, Williams
IgA deficiency
Mass screening

*CD, celiac disease*.

### Screening for celiac disease in high-risk groups

Because of the high prevalence of CD among these groups, evidence-based guidelines recommend screening for early detection of the disease (7) (Table 8). A plethora of studies are available on 2 of the populations who belong to these high-risk groups, namely first-degree relative of patients with CD and children with type 1 diabetes mellitus (T1DM).

#### First-degree relatives of CD patients

Many studies have demonstrated that first-degree relatives (FDRs) of celiac patients have a higher risk of developing CD than the general population, with a prevalence ranging from 1.6 to 38% (91). Based on a systematic review and meta-analysis, Sing et al. (91) reported that the pooled prevalence of CD was 7.5% in 10,252 FDRs (91). The risk of developing CD among FDRs is influenced by gender and HLA haplotype (15, 16). CD occurs more often in girls (female: male ratio of 2–3:1), and HLA-DQ2 homozygous children have a significantly higher risk of developing CD than HLADQ2 heterozygous children (14.9 vs. 3.9%, respectively, at the age of 3 years) (15).

#### Children with conditions/diseases associated with CD

The prevalence of CD in patients with T1DM has been reported by most studies as ranging between 4 and 10% (92). Many children with T1DM and CD are asymptomatic or at least symptoms of CD have not been observed. In these cases, CD may only be detected by serologic screening. However, it has been shown that strict adherence to a GFD was < 30% in children with both CD and T1DM, compared to 81% among patients with CD only (93). Maintaining a strict GFD in addition to a diabetic diet requires additional time, effort, and expense. Evidence is inconclusive as to whether the benefits of screening and potentially treating asymptomatic individuals outweigh the harms of managing a population already burdened with a serious illness. The Celiac Disease and Diabetes-Dietary Intervention and Evaluation Trial (CD-DIET) (ClinicalTrials.gov Identifier: NCT01566110) involves screening of children and adults with T1DM for asymptomatic CD, followed by randomization to a GFD or no-GFD group, to assess outcomes (including diabetes control, bone mineral density, and health-related quality of life) over 1 year to clarify effects of screening and treating asymptomatic CD in this population with a GFD (94).

### Mass screening

Screening the general population, also called mass screening, would theoretically be the best form of secondary prevention since it could potentially detect all cases of CD, including those in asymptomatic patients as well as those in patients who lack symptoms. Results from most screening studies performed in the general population suggest that symptoms are not reliable predictors of CD (15, 95, 96), reinforcing the place of mass screening as the best strategy for secondary prevention of CD enabling early treatment to reduce the burden of morbidity and mortality associated with untreated CD (97, 98).

However, mass screening for CD is still debated, partly because evidence has been lacking on the accuracy of diagnostic tests and on the health benefits after diagnosis and treatment of asymptomatic detected patients. This uncertainty also affects the cost benefit of mass screening, which is needed for implementation of mass screening for CD (99, 100). Most studies on the diagnostic accuracy of diagnostic tests for CD have been conducted in symptomatic patients (101, 102). Because the positive predictive value declines when the test is used in settings with a low pre-test prevalence, such as the general population, the sensitivity and specificity of these tests are lower in the setting of mass screening.

Recently, a prospective study performed in Rotterdam has shown a positive predictive value of 81% for CD in the general pediatric population (96). These authors also showed that undiagnosed CD is associated with a lower body mass index compared to controls at the age of 9 years (96) and associated with fetal growth restriction and lower birth and placental weight during pregnancy (8). Additional information about the importance and effectiveness of screening comes from a population-based-screening study performed in Sweden; this study showed that at 10 years of age, children with CD detected by screening already had reduced bone mineral density in the total body and spine compared with age-matched controls. These differences were not found in children with CD on a GFD from 3 years of age, indicating that children with screening-detected CD benefit from early diagnosis and treatment (103).

The data on the benefits and harms of screening are limited. Only one randomized trial evaluated the effectiveness of GFD vs. no GFD in apparently asymptomatic adults with screen-detected CD and found that initiation of a GFD in screen-detected adults with unrecognized symptoms was associated with improved gastrointestinal symptoms (104).

Other traditional reason against mass screening are that adherence to the GFD in minimally or asymptomatic patients would be lower than in symptomatic patients and that the quality of life is decreased in screening-detected CD patients following a GFD. However, 10 years of follow up among Dutch children and the results of a sub-study from the ETICS project showed similar adherence rates to the GFD in screening-detected children compared with clinically detected children (105, 106). No significant differences in health-related quality of life (HRQoL) were observed between screening-detected and symptom-detected adult patients (107, 108). Furthermore, a systematic review and meta-analysis on dietary adherence and HRQoL in adult patients with CD detected by screening showed a significantly lower HRQoL after 1 year of treatment with a GFD in symptom-detected patients compared to screening-detected patients (109). Despite the aforementioned literature that is positive about screening of the general population, the current literature recommending mass screening is limited.

## Tertiary prevention

### Gluten-free diet (GFD)

Tertiary prevention focuses on reducing the impact of existing disease by improved treatment (Table 9). One of these strategies involves optimizing adherence to the GFD. Complete removal of gluten from the diet is a challenge, as gluten is present in a wide variety of foods. However, since the introduction of allergen labeling in the European Union (EU) in 2005, gluten cannot be hidden in products. The amount of gluten capable of initiating an antigenic reaction has been estimated to be >20 mg/kg (or parts per million = ppm) of gluten, and contamination below 20 ppm is considered safe over a wide range of foods in daily consumption.

**Table 9:** Tertiary prevention strategies for celiac disease.

**Strategy**	**Successful**
Optimal adherence to the GFD	Yes
Treatment options for CD other than a GFD
Larazotide acetate	Unclear
Endopeptidases
Latiglutenase (ALV003)	Unclear
Aspergillus niger prolyl endoprotease (AN-PEP)	Unclear
Desensitization therapy (therapeutic vaccine)	Unknown

*CD, celiac disease; GFD, gluten-free diet*.

### Improving monitoring of and adherence to the GFD

#### Dietitian

Due to the complexity of the GFD, it is essential that newly diagnosed patients be referred to a dietitian with expertise in CD. A delay in referral, or no referral at all, increases the likelihood of the patient obtaining inaccurate information from the Internet, health food stores, alternative health practitioners, family, friends, and other sources, often resulting in confusion, frustration, and insufficient knowledge regarding CD and the GFD (110).

Gluten-containing cereals, such as wheat, barley, and rye, are important sources of dietary iron, calcium, folate, and vitamin B12. As the treatment of CD with a GFD can lead to nutritional deficiencies, the support of a dietitian is necessary to avoid these deficiencies. Also, consultation with someone with knowledge in the field of replacement (gluten-free) products, such as amaranth, buckwheat, quinoa, sorghum and teff, is of great importance and could improve intakes of protein, iron, calcium, and fiber by patients with CD (111).

#### Validated food questionnaires

A dietary interview to assess compliance with the GFD is the best way to detect errors in GFD adherences among children and young adults, but it is timue-consuming (20–30 min per patient) and requires expert personnel. Several short questionnaires have been developed to measure GFD adherence in order to save time, and while some are not sensitive enough, others are useful in assessing compliance to the diet (112). With the increasing use of self-assessment and alternative follow-up methods for CD patients, including electronic patient records and E-health tolls, completing questionnaires before or during a medical consultation should be easily implemented in the healthcare of children and young adults with CD (113).

#### Measurement of gluten immunogenic peptides (GIPs)

Available methods to assess GFD compliance are time-consuming and are also insufficiently sensitive to detect occasional dietary transgressions that may cause gut mucosal damage. Determination of serum TG2A is usually used during the follow-up of a patient on a GFD, as this marker improves with gluten elimination (114). However, it has been reported that even while following a GFD, children and women with CD have a much higher prevalence of gastrointestinal symptoms than controls, and they also use healthcare services more often (115). As mucosal damage may still persist without TG2A, antibody testing may be negative in patients with only partial adherence to the GFD (116). Therefore, it is necessary to have a non-invasive biomarker to monitor compliance with the GFD. Certain GIPs are resistant to gastrointestinal digestion and can interact with the immune system of patients with CD to trigger an autoimmune response against transglutaminase and other antigens. A proportional fraction of the GIPs absorbed in the gastrointestinal tract make it to the circulation and are excreted in urine. GIPs are detectable in concentrated urines and may be useful in clinical practice as a monitoring tool to follow-up compliance with the GFD. GIPs are detected in urine samples 6–48 h after gluten intake (>25 mg) and remained detectable for 1–2 days (117).

### Treatment options for CD other than the GFD

Several other treatments aimed at different pathogenic targets of CD have been studied in recent years: modification of gluten to produce non-immunogenic gluten, endoluminal therapies to degrade gluten in the intestinal lumen, increasing gluten tolerance, modulation of intestinal permeability, and regulation of the adaptive immune response. However, not all of these therapies have been tested in clinical trials (yet). The most advanced studies are devoted to larazotide acetate and prolyl-endopeptidases degrading toxic gluten peptides and to therapeutic vaccination.

#### Larazotide acetate

Patients with active CD have increased intestinal permeability. Zonulin, a modulator of epithelial tight junctions, is overexpressed in these patients. Release of zonulin in response to binding between gliadin peptides and a specific chemokine receptor (CXCR3) results in a measurable reduction in the usual intestinal barrier and allows enhanced passage of gliadin. This mechanism has been the target of advanced research that led to the development of larazotide acetate (AT-1001), an octapeptide that inhibits gliadin-induced intestinal permeability. Several phase I and II clinical trials have confirmed the safety of this agent and suggest a potential beneficial effect of larazotide (118, 119). Additionally, patients who were treated with larazotide acetate had significantly fewer symptoms (patient reported Celiac Disease Gastrointestinal System Rating Score) compared with those taking a placebo (120–122). A dose-response effect was not seen, with the most benefit encountered at the lowest (0.5 mg) of 4 dosages administered (121); however, this study did not measure histologic endpoints, and larazotide had no significant effect on serologic levels of specific CD antibodies as TG2A.

#### Endopeptidases

The gluten peptides, which are responsible for inducing the immunological response in CD patients, are rich in proline and are highly resistant to enzymatic proteolysis within the digestive tract. For many years, there have been studies conducted to investigate the effectiveness of orally administered prolyl oligo-peptidases in the degradation of toxic gliadin peptides before they reach the mucosa of the small intestine.

Latiglutenase (ALV003, Alvine Pharmaceuticals, San Carlos, CA, USA) is an orally administered mixture of 2 recombinant gluten-specific proteases—a cysteine protease (EP-B2) and a prolyl endopeptidase (PEP)—which have been shown *in vitro* to degrade gluten (123). Both endopeptidases are active and stable at gastric pH (124). In a Phase 2 study with ALV003, adults with biopsy-proven CD were randomly assigned to groups receiving ALV003 or placebo, together with a daily 2 g gluten challenge. Upper endoscopy was performed at baseline and after the gluten challenge. Primary endpoint included the villus height to crypt depth ratio and CD3^+^ intra-epithelial lymphocytes (IEL) density. Serologic markers and symptoms were also assessed. In the ALV003 group, there were no changes in histological measures, while in the placebo group, evidence of mucosal injury was shown after gluten challenge. In contrast, no differences were seen in symptoms and serologic markers of CD in both groups. In a phase 2 study involving patients with symptomatic CD and histologic evidence of significant duodenal mucosal injury, ALV003 did not improve histologic and symptom scores when compared with placebo (125). However, a subgroup-analysis of the study showed a statistically significant, dose-dependent reduction in the severity and frequency of symptoms in seropositive but not in seronegative patients (126).

Aspergillus niger prolyl endoprotease (AN-PEP; DSM, Heerlen, The Netherlands) is also an endopeptidase, isolated from the fungus Aspergillus niger. The enzyme is active between a pH of 2 and 8, with an optimum activity at pH 4–5, thus, in the stomach and small intestine (127). In a randomized, placebo-controlled, crossover study, 18 self-reported gluten-sensitive subjects consumed a porridge containing 0.5 g gluten together with two tablets containing either a high or low dose of AN-PEP or placebo. Gastric and duodenal contents were sampled over 180 min. The primary outcome was defined as the efficacy of the high dose of AN-PEP compared with placebo in degrading at least 50% of gluten, based on the amount of gluten detected in the duodenum. The researchers concluded that the AN-PEP enzyme is effective in degrading small amounts of gluten as part of a complex meal in the stomach, but it is not intended to replace a GFD in patients with gluten-related disease (128). In a Phase 2a double-blinded, placebo-controlled, randomized trial, 16 CD patients on a GFD, who were in serological and histopathological clinical remission, were administered AN-PEP or a placebo with gluten-containing toast (~7 g/day gluten). The mean score for the gastrointestinal subcategory of the CD quality (CDQ) was relatively high throughout the study, indicating that AN-PEP was well-tolerated. In the efficacy phase, the CDQ scores of patients consuming gluten with placebo or gluten with AN-PEP did not significantly deteriorate and, moreover, no differences between the groups were observed.

Larazotide and PEPs will not become an alternative to the GFD and their potential role as therapeutic agents for CD remain unclear.

### Desensitization therapy (therapeutic vaccine)

NexVax2 from ImmusanT is a desensitizing vaccine that uses three dominant gluten peptides administered subcutaneously to induce an immune response in CD patients who carry the immune recognition gene HLA-DQ2.5, which accounts for disease in 80–90% of patients. The aim of the vaccine is to use peptide-based immunotherapy to shift the T-cell response from pro-inflammatory to regulatory, in order to restore immune tolerance to gluten. Phase 1b clinical trials of this vaccine have recently been completed supporting the safety, tolerability and relevant bioactivity of Nexvax2 (129).

## Conclusions

- Celiac disease is a common autoimmune disorder induced by ingestion of gluten in genetically susceptible individuals.
- Only a minority of those who are at genetic risk develop the disease.
- The incidence of CD has increased over the last half-century, resulting in rising interest in identifying risk factors for CD to enable prevention.
- Environmental and/or lifestyle factors play a causal role in the development of CD.
- For primary prevention (i.e., interventions before CD occurs), early feeding practices seem to have no impact on the risk of developing CD during childhood. Other environmental influences have been investigated as potential risk factors; however, they have not yet led to primary prevention strategies.
- Secondary prevention is possible through early diagnosis and treatment; however, it will not identify all CD patients as long as mass screening has not been introduced.
- As a gluten-free diet is a major challenge, tertiary prevention strategies are under evaluation; however, none of these measures are currently recommended as treatment.

## Author contributions

CM: Corresponding author, primary responsibility for communication with the journal during the manuscript submission, drafting the work, final approval of the version to be published, agreement to be accountable for all aspects of the work. RS: Critical revision of the article, final approval of the version to be published, agreement to be accountable for all aspects of the work. HS: Critical revision of the article, final approval of the version to be published, agreement to be accountable for all aspects of the work. LM: Drafting the work, supervising corresponding author, final approval of the version to be published, agreement to be accountable for all aspects of the work.

### Conflict of interest statement

The authors declare that the research was conducted in the absence of any commercial or financial relationships that could be construed as a potential conflict of interest.
